# Correlation between chemical composition and antibacterial activity of some Lamiaceae species essential oils from Tunisia

**DOI:** 10.1186/s12906-020-02888-6

**Published:** 2020-04-03

**Authors:** Sarra Moumni, Ameur Elaissi, Amine Trabelsi, Abderrahmen Merghni, Imed Chraief, Brahim Jelassi, Rachid Chemli, Salima Ferchichi

**Affiliations:** 1 Laboratory of Chemical, Pharmaceutical and Pharmacological Drug Development LR12ES09, Faculty of Pharmacy, University of Monastir, rue Avicenne, 5000 Monastir, Tunisia; 2 Laboratory of Pharmacognosy, Faculty of Pharmacy, University of Monastir, Avenue Avicenne, 5000 Monastir, Tunisia; 3 Laboratory of Antimicrobial Resistance LR99ES09, Faculty of Medicine of Tunis, University of Tunis El Manar, Tunis, Tunisia; 4 Department of Biochemistry, Faculty of Medicine, University of Monastir, Rue Avicenne, 5000 Monastir, Tunisia; 5 Laboratory of Transmissible Diseases and Biologically Active Substances LR99ES27, Faculty of Pharmacy, University of Monastir, rue Avicenne, 5000 Monastir, Tunisia; 6 Biochemistry Laboratory CHU Farhat Hached, 4000 Sousse, Tunisia

**Keywords:** Lamiaceae family, Essential oils, Chemical composition, Antibacterial activity, Principal component analysis (PCA), Hierarchical cluster analysis (HCA)

## Abstract

**Background:**

Lamiaceae family is one of the most diverse and common plant families in terms of ethnomedicine due to their potential therapeutic effects. The aim of this study is to investigate the correlation between the chemical composition and the antibacterial effect of five essential oils from this family against five reference bacterial strains responsible of nosocomial diseases and foodborne illnesses.

**Methods:**

The commercial essential oils of Tunisian *Rosmarinus officinalis*, *Thymus capitatus*, *Origanum majorana* and *Salvia officinalis* were analyzed by GC/FID and GC-MS. Essential oils were evaluated for their antibacterial activities by disc diffusion and microbroth dilution methods against five reference bacterial strains: *Pseudomonas aeruginosa*, *Escherichia coli*, *Salmonella enterica*, *Bacillus subtilis* and *Staphylococcus aureus*. The inhibition zone diameter values and the twenty major compounds of the selected essential oils were subjected to PCA and HCA analysis.

**Results:**

Analysis by GC/FID and GC/MS allowed the identification of ninety-one components representing 96.0 to 98.2% of the total oils. The different component contents varied according to the species. The main components were carvacrol, *1,8*-cineole, *α*-thujone, *α*-terpineol and *α*-pinene. The PCA and the HCA of the selected essential oil components and the inhibition zone diameter (IZD) values identified four species groups and subgroups. Each essential oils group constituted a chemotype responsible for their bacterial inhibition ability. *Thymus capitatus* essential oil showed the strongest antibacterial activity with MBC ranging from 0.73 to 2.94 mg mL^− 1^.

**Conclusion:**

*Rosmarinus officinalis*, *Thymus capitatus*, *Origanum majorana* and *Salvia officinalis* essential oils have shown promising antibacterial activities against reference bacterial strains responsible for nosocomial diseases and foodborne illnesses.

## Background

In the last few years, the interest in natural medicine has been increasing in industrialized societies particularly against microbial agents because of the ever growing problem of antibiotic resistance [1]. Natural products are an important source of novel and chemically diverse molecules for drug discovery [2]. In fact, because of their bioactive compounds and their antimicrobial properties, the interest in medicinal plants has increased considerably [3]. Actually, 69% of currently employed antibiotics are derived from natural products [4]. Essential oils are volatile fractions obtained from medicinal and aromatic plants, they have been screened for their potential uses as alternative remedies for the treatment of many infections and as natural food preservatives [5–8]. Lamiaceae family is one of the most diverse and widespread plant families in term of ethnomedicine and its medicinal value is mainly based on the volatile oils composition [9]. It was reported that the leaves of *T. capitatus* were used for many purposes such as unguents for embalming by Egyptians [10], antispasmolytic, antiseptic, expectorant, anti-ulcers, anti-dermatitis and rheumatic pains [11]. *T. capitatus* showed good results in treatment of skin diseases and as tonic and antitussive agent [12]. *S. officinalis* is used for its antibacterial [13, 14], antiviral [15] and antioxidant [16] activities. It is also used as a flavoring agent in perfumery and cosmetics [17]. *R. officinalis* has been traditionally used for both medical and culinary purposes [18]. Indeed, the whole fresh plant or its dried milled powder is used as a food flavoring and preservative agent [19]. Likewise, *R. officinalis* is known as a medicinal herb with significant activities against many illnesses especially against headaches, respiratory diseases and several neuropsychiatric disorders, according to traditional Tunisian medicine [20]. *O. majorana* L. is used as a medicinal plant; it has traditionally been used as stimulant, condiment, and tonic [21]. In addition, *O. majorana* L. essential oil is used for its antibacterial [22] and antioxidant activities [21].

Because of their interesting physicochemical characteristics of substantial value, the Lamiaceae essential oils have garnered research and industrial interests for their use as natural products [23]. However, in Tunisia, apart from *R. officinalis* EO, Lamiaceae EOs production is still weak despite the plants abundance and scientific researches on these EOs bioactivities [14, 24, 25]. Essential oils chemical composition is affected by several factors, including the species, part of the plant, season of harvesting, geographical origin, and also the extraction method and conditions which consequently impact on their bioactive properties [26–29]. Hence, the present work aimed to study the chemical composition of five commercial Lamiaceae EOs from Tunisian provenance and their antibacterial effects against five pathogen bacteria models. A correlation between 20 major compounds and their antibacterial ability was investigated.

## Methods

### Essential oils

EO samples were offered by three Tunisian companies specialized in EO extraction: Bio Orient (*S. officinalis* and *T. capitatus*), Agriland (*R. officinalis*_1_ and *O. majorana*) and Uticafloria (*R. officinalis*_2_). *S. officinalis* was collected from Sfax (34°44′N, 10°46′E) in the southeast of Tunisia. *T. capitatus* was harvested from Sejnane (37°03′23″N, 9°14′18″E) in the northwest of Tunisia. *O. majorana* was collected from Zaghouan (36°24′N, 10°09′E) in the north of Tunisia. *R. officinalis*_*1*_ was collected from Sbikha (35°56′N, 10°01′E) in the center of Tunisia. *R. officinalis*_*2*_ was harvested from El Fahs (36°23′N, 9°54′E) in the north of Tunisia. Botanical voucher specimens have been identified by Professor Ameur ELAISSI and deposited with the herbarium of the Pharmacognosy Loboratory in Faculty of Pharmacy, Monastir, Tunisia, under the following references: 0181, 0182, 0183, 0184 and 0185.

The essential oils were extracted by hydrodistillation of fresh aerial parts. For each species, 3 Samples of the obtained EO were stored at 4 °C until analysis was attempted.

### Chemical analysis

Quantitative and qualitative data of all the essential oils were determined in triplicate by GC/FID and GC/MS respectively.

### Gas chromatography analysis

One microliter sample of 10% solution of essential oil in purified hexane was injected for analysis using a HP 6890 chromatography apparatus equipped with flame ionization detector (FID) and HP5 column (30 m × 0.25 mm, film thickness 0.25 μm). The analytical conditions were as follows: injector and detector temperature were maintained at 250 °C and 280 °C respectively. Oven temperature was programmed to rise from 60 °C to 250 °C at 5 °C/min, isothermal temperature 60 °C for 1 min and 250 °C for 3 min; carrier gas was N_2_ with a flow rate of 1.2/min. The software HP Chemstation, allowing assimilation of the percentages of the peak areas to the percentages of the various constituents was used to calculate relative concentration. Kovats retention Indexes (KI) were obtained by running a series of aliphatic hydrocarbons (C9-C28) increasing number order of carbon atoms on the column.

### Gas chromatography-mass-spectrometry analysis

The essential oils analysis was performed using a Hewlett Packard (HP) 5890 II GC equipped with a HP-5MS capillary column (30 m × 0.25 mm, film thickness 0.25 μm) and a HP 5972 mass selective detector. Helium was used as the carrier gas at a flow rate of 1.2 ml/min. For GC/MS detection, an electron ionization system, with ionization energy of 70 eV, a scan time of 1.5 s and mass range 40–300 amu was used. The GC/MS parameters were identical to those for the GC/FID analysis.

### Compound identification

The compounds were identified by comparing retention indices (determined relatively to the retention time of aliphatic hydrocarbons (C9-C28)) of the mass spectra with those of authentic compounds by means of Wiley 275.L, 6th edition mass spectral library and NBS75K.L data bases. The identification is confirmed by comparing their retention indices with data published in the literature [30, 31].

### Antibacterial assay

#### Bacterial strains

Three Gram negative bacteria (Gram (−)), *Pseudomonas aeruginosa* (CIP 82118), *Salmonella enterica* (CIP 80.39), *Escherichia coli* (CIP 53.126) and two Gram positive (gram (+)) bacteria *Staphylococcus aureus* (CIP 4.83) and *Bacillus subtilis* (CIP 52.62) were used to carry out this study. Microorganisms were obtained from the culture collection of the laboratory of transmissible diseases and biologically active substances, Faculty of Pharmacy, Monastir, Tunisia.

#### Agar disk diffusion method

To evaluate the antibacterial activity of the selected EOs, the agar disk diffusion assay [32] was carried out. Overnight cultures (24 h at 37 °C) in MH broth were prepared. The bacterial inocula were obtained by diluting the overnight cultures in Mueller Hinton Broth medium to adjust the optical density at approximately 0.5 Mc Farland standards. Absorbent discs (Whatman disc No.3, 6 mm diameter) were impregnated with 10 μL of essential oil and then placed on the surface of inoculated Petri dishes. Positive control discs of standard antibiotic GENTAMICIN® (10 μg /disc) were tested. After 24 h of incubation at 37 °C, the inhibition zones were measured and expressed in mm. All experiments were performed in triplicate.

#### Minimum inhibitory and minimum bactericidal concentrations

For each essential oil, the minimum inhibitory concentration (MIC) and minimum bactericidal concentration (MBC) values against the bacterial strains were determined according to the National committee for clinical Laboratory Standard [33]. An overnight culture (37 °C) in Mueller Hinton broth medium of the tested strains was prepared by adjusting the turbidity of each bacterial culture to reach an optical density of 0.5 Mc Farland standards.

The broth dilution method was carried out in 96-well microtitre plates using bacterial reference strains. To obtain stable diffusion, EOs stock solutions were prepared aseptically in DMSO (80, 20%, v/v), transferred to sterile 96-well microtitre plates containing the Mueller Hinton medium and then diluted by two-fold serial dilution. Ten microliter of each strain inoculum was added to each well. Negative control wells contained only bacteria in the MH broth medium. Positive control wells contained 10 μg mL^− 1^ Gentamicin® antibiotic. After incubation for 24 h at 37 °C, 10 μL of 0.02% (p/v) resazurin solution was added. Bacterial growth was evaluated by the observation of the wells color change from blue to pink. The MIC was defined as the lowest concentration that completely inhibit visible cell growth during a 24 h incubation at 37 °C (blue colored). DMSO 20% (v/v) had no inhibition effect. To determine the minimum bactericidal concentration (MBC) values, 10 μL of each well with no visible growth were removed and inoculated in MH plates. After 24 h of incubation at 37 °C, the number of surviving organisms was determined. MBC was defined as the lowest concentration at which 99% of the bacteria were killed. The experiments were performed in triplicate.

### Statistical analysis

The data were subjected to statistical analysis using analysis of variance (ANOVA), and the significance of the difference between means was assumed at *p* ≤ 0.05 using Student Newman and Keuls (SNK) test. Twenty Compounds detected in the essential oil samples at an average concentration greater than 3.0% were selected and used to evaluate whether the identified compounds may be useful in reflecting chemotaxonomic and biological activities relationships. Data of antibacterial activity and components were subjected to principal components (PCA) and hierarchical cluster analysis (HCA). Statistical tests were performed using IBM® SPSS® 20 software (SPSS INC.Chicago,IL, USA).

## Results

### Chemical composition

The essential oils of *T. capitatus*, *S. officinalis*, *R. officinalis*_1_, *R. officinalis*_2_, and *O. majorana* were subjected to the chromatographic analyses (GC/FID and GC/MS). Ninety-one components representing 96.0 to 98.2% of the total oils content were identified (Table 1). The identified components were divided in to eleven chemical classes (Table 1). The monoterpene phenols (1.02 ± 0.4–56.6 ± 2.8%) constituted the main class with carvacrol (tr – 56.1 ± 3.0%), as major constituent of *T. capitatus* EO. The class with the second highest contents was composed of monoterpene ketones (1.7 ± 2.8–48.9 ± 4.1%). *S. officinalis* essential oil was characterized by the highest content in this class, with *α*-thujone (0.1 ± 0.0–22.8 ± 1.5%) having the highest content in this species, followed by camphor (1.61 ± 2.7–19.3 ± 2.1%) and *β*-thujone (tr - 6.8 ± 0.5%). Verbenone (tr – 3.7 ± 0.5) have the highest content in *O. majorana*. The monoterpene oxides (2.9 ± 4.6–47.2 ± 0.5%) represented the third major class, constituted by *1,8*-cineole (2.9 ± 4.6–47.2 ± 0.5%), *R. officinalis*_1_ have the highest content while *T. capitatus* have the smallest one. The fourth major class was composed of monoterpene hydrocarbons (17.5 ± 6.6–40.9 ± 5.2%), *O. majorana* and *R. officinalis*_*2*_ were characterized by the highest mean percentages of this class (40.9 ± 5.2 and 36.8 ± 9.7%, respectively). *α*-pinene (0.8 ± 0.0–19.4 ± 5.4%) have the highest content in the essential oils of *R. officinalis*_1_, *R. officinalis*_2_ and *S. officinalis*. The second major component in this class, *ϒ*-terpinene (0.3 ± 0.0–16.3 ± 1.5%), have the highest content in *O. majorana* and *T. capitatus*, these two species are characterized by the highest content of *p*-cymene (0.9 ± 1.0–9.7 ± .8%). *O. majorana* is characterized by the highest content of *α*-terpinene (0.1 ± 0.1–7.7 ± 1.3%) however *R. officinalis*_2_ have the highest content of camphene (0.2 ± 0.0–6.5 ± 1.6%). The major components of the fifth monoterpene alcohols class (4.6 ± 1.0–39.6 ± 3.1%), were *α*-terpineol (0.1 ± 0.0–27.4 ± 2.8%) and borneol (1.0 ± 0.8–5.4 ± 1.5). The sixth class was sesquiterpene hydrocarbons (2.5 ± 0.6–10.0 ± 3.3%) represented essentially by *(E)*-caryophyllene (1.7 ± 0.5–5.2 ± 0.9%) which was more abundant in *R. officinalis EO* samples, and *α*-humulene (0.1 ± 0.0–4.8 ± 2.0%), characterizing *S. officinalis* EO.

**Table 1:** Chemical composition of *S. officinalis*, *T. capitatus*, *R. officinalis*_1_, *R. officinalis*_2_ and O. majorana essential oils

	*KI*^*a)*^	Composition [%]				
		*S. officinalis*	*T. capitatus*	*R. officinalis*_*1*_	*R. officinalis*_*2*_	*O. majorana*
Tricyclene	923	0.5	-^b)^	0.1	0.2	–
*α*-Thujene	927	0.4	1.0	0.1	0.1	1.0
*α*-Pinene	934	6.2	0.8	7.0	19.4	1.1
Camphene	949	6.2	0.2	2.7	6.5	0.2
Sabinene	974	0.1	–	0.1	Tr^c)^	2.2
*β*-Pinene	978	2.6	0.5	4.6	3.6	0.6
*β*-Myrcene	992	0.8	1.9	–	0.1	1.5
*α*-Phellandrene	1002	Tr	0.3	–	1.8	0.4
*δ*-3-Carene	1006	Tr	0.1	0.2	0.2	0.2
*α*-Terpinene	1012	0.1	2.0	0.1	0.4	7.7
*p*-Cymene	1025	2.1	7.4	1.9	0.9	9.7
Limonene	1029	1.6	0.5	–	2.5	–
*1.8*-Cineole	1032	10.3	2.9	47.2	37.6	4.0
*(Z)-β*-Ocimene	1038	Tr	Tr	Tr	0.1	–
*(E*)- *β-*Ocimene	1048	Tr	0.1	Tr	0.1	–
*ϒ*-Terpinene	1058	0.6	10.8	0.3	0.5	16.3
*(Z)*-Sabinene hydrate	1068	Tr	0.6	0.1	–	1.9
*(Z)*-Linalool oxyde furanoid	1070	–	Tr	–	–	–
*α*-Terpinolene	1090	0.3	0.2	0.3	0.3	Tr
*(E)*-Sabinene hydrate	1098	–	1.1	–	–	3.7
Linalool	1101	–	0.5	1.0	0.5	2.8
*(E)-4*-Thujanol	1104	–	–	–	–	1.1
*α*-Thujone	1109	22.8	–	Tr	0.1	–
*β*-Thujone	1119	6.8	–	0.1	Tr	–
*(Z)*-*2*-Menth-*2*en-ol	1124	–	–	–	–	0.8
α-Campholenal	1127	Tr	–	Tr	Tr	–
*(E)*-Menth-*2*-en-ol	1142	–	–	–	–	0.8
Camphor	1146	19.3	1.6	13.3	7.1	Tr
Isoborneol	1158	–	–	Tr	Tr	–
Pinocarvone	1160	–	Tr	–	–	–
Borneol	1167	4.0	1.0	5.4	4.4	–
*(Z)*-Pinocamphone	1175	Tr	–	–	–	–
Terpinen-*4*-ol	1178	0.4	0.8	1.0	0.5	0.3
*p*-Cymen-*8*-ol	1186	0.1	Tr	0.1	Tr	–
*α*-Terpineol	1192	0.1	0.2	2.6	1.6	27.4
Myrtenal	1198	0.1	–	–	–	–
Myrtenol	1205	Tr	–	–	–	0.3
*(E)*-Dihydrocarvone	1206	–	Tr	–	–	–
Verbenone	1207	–	–	Tr	Tr	3.7
*(Z)*-Sabinene hydrate acetate	1211	–	–	–	–	0.4
Compound class and name						
endo-Fenchyl acetate	1221	Tr	–	–	–	–
Nerol	1229	–	–	–	–	Tr
*(Z)*-Carveol	1230	0.1	–	–	–	–
Thymol methyl ether	1233	–	Tr	–	–	–
Carvacrol methyl ether	1246	–	Tr	–	–	–
Linalyl acetate	1258	0.1	–	–	–	–
*(E)*-Sabinene hydrate acetate	1260	–	–	–	–	2.0
Geraniol	1260	–	0.3	–	–	0.5
Geranial	1278	–	0.1	–	–	–
Bornyl acetate	1287	1.6	–	0.9	0.2	–
Thymol	1294	–	0.4	–	Tr	0.6
Carvacrol	1304	Tr	56.1	–	–	0.6
Myrtenyl acetate	1325	–	–	–	–	1.2
*α*-Cubebene	1351	0.1	Tr	–	–	–
Eugenol	1357	–	Tr	–	–	–
Thymol acetate	1359	–	Tr	–	–	–
Carvacrol acetate	1368	–	0.2	–	–	–
*α*-Ylangene	1373	–	–	0.4	0.1	–
*α*-Copaene	1374	–	0.1	–	–	–
Geranyl acetate	1385	–	–	–	0.4	Tr
*β*-Elemene	1386	Tr	–	–	–	–
*β*- Cubebene	1389	Tr	Tr	–	–	–
*(E)*-Caryophyllene	1420	3.9	4.2	5.2	5.0	1.7
*β*-Gurjunene	1425	–	Tr	–	–	–
*(E)-α*-Bergamotene	1429	–	Tr	–	–	–
Aromadendrene	1429	Tr	0.1	0.4	Tr	–
*α*-Humulene	1455	4.8	0.2	0.3	0.7	0.1
*allo*-Aromadendrene	1462	Tr	–	0.2	Tr	–
*ϒ*-Muurolene	1475	Tr	Tr	–	–	–
Germacrene-*D*	1482	Tr	–	0.2	–	–
*β*-Selinene	1487	Tr	–	–	–	0.5
Viridiflorene	1493	–	Tr	–	–	–
Bicyclogermacrene	1495	–	–	0.1	–	0.2
Valencene	1495	–	Tr	–	–	–
*α*-Muurolene	1496	0.1	–	0.1	0.3	–
*α*-Amorphene	1500	–	–	–	0.1	–
*(Z)-α*-Bisabolene	1502	Tr	–	–	–	–
*β*-Bisabolene	1506	Tr	0.1	–	–	–
*ϒ*-Cadinene	1517	0.1	Tr	–	–	Tr
*δ*-Cadinene	1525	0.1	Tr	0.3	0.6	–
*α*-Cadinene	1536	0.1	–	–	–	–
*α*-Calacorene	1543	Tr	0.1	–	–	–
Germacrene-*B*	1551	0,6	–	–	–	–
*β*-Calacorene	1558	0,1	–	–	–	–
Spathulenol	1579	0,1	0,4	–	–	0,1
Caryophyllene oxide	1584	0,5	0,3	0,2	0,1	0,4
Vidiflorol	1593	Tr	–	–	Tr	–
*epi*-*α*-Cadinol	1640	Tr	Tr	–	–	–
τ-Muurolol	1644	Tr	–	–	–	–
*α*-Muurolol	1652	Tr	–	–	–	–
*α*-Cadinol	1656	Tr	–	–	–	–
Monoterpene hydrocarbons		21.6 ± 6.1^ab^	25.7 ± 5.5^abc^	17.5 ± 6.6^a^	36.8 ± 9.7^bc^	40.9 ± 5.2^c^
Monoterpene oxides		10.3 ± 1.4^b^	2.9 ± 4.6^a^	47.2 ± 0.5^d^	37.6 ± 0.5^c^	4.0 ± 0.5^a^
Monoterpene alcohols		4.7 ± 0.3^a^	4.6 ± 1.0^a^	10.2 ± 2.8^b^	7.0 ± 2.1^ab^	39.6 ± 3.1^c^
Monoterpene ketones		48.9 ± 4.1^c^	1.7 ± 2.8^a^	13.5 ± 2.9^b^	7.2 ± 1.3^a^	3.7 ± 0.5^a^
Monoterpene aldehydes		0.1 ± 0.0^a^	0.1 ± 0.1^a^	Tr	Tr	–
Monoterpene esters		1.7 ± 0.2^b^	0.2 ± 0.1^a^	0.9 ± 0.2^a^	0.6 ± 0.3^a^	3.6 ± 0.6^c^
Monoterpene ethers		–	0.1 ± 0.1a	–	–	–
Monoterpene phenols		Tr	56.6 ± 2.8^b^	Tr	–	1.2 ± 0.4^a^
Sesquiterpene hydrocarbons		10.0 ± 3.3^b^	4.9 ± 0.3^ab^	7.1 ± 1.0^ab^	7.0 ± 4.3^ab^	2.5 ± 0.6^a^
Sesquiterpene alcohols		0.3 ± 0.2^a^	0.4 ± 0.3^a^	–	Tr	0.1 ± 0.1^a^
Sesquiterpene oxides		0.5 ± 0.3^a^	0.3 ± 0.4^a^	0.2 ± 0.3^a^	0.1 ± 0.1^a^	0.4 ± 0.1^a^
Not identified		1.8 ± 0.5^a^	2.8 ± 0.8^ab^	3.4 ± 0.3^ab^	3.6 ± 1.4^ab^	0.4 ± 0.5^b^
Total identified		98.2	97.2	96.6	96.4	96.0

^a^Kovats retention index determined on a HP5 capillary column. ^b^-: not detected. ^c^*Tr* Trace (< 0.1%)

**Fig. 1:**
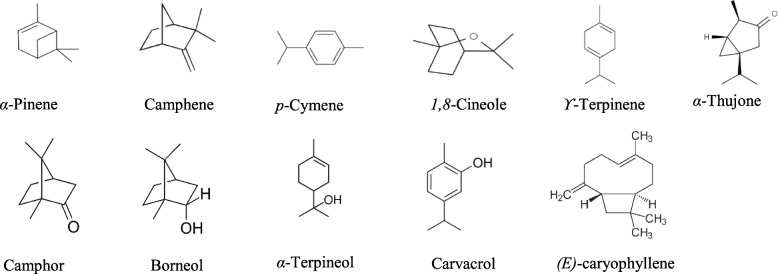
Chemical structures of some compounds isolated from *S. officinalis*, *T. capitatus, R. officinalis*_1_, *R. officinalis*_2_ and *O. majorana *Essential Oils

Figure 1 shows the chemical structures of some of the main components.

### Principal component analysis (PCA) and hierarchical cluster analysis (HCA)

#### Chemical classes

**Fig. 2:**
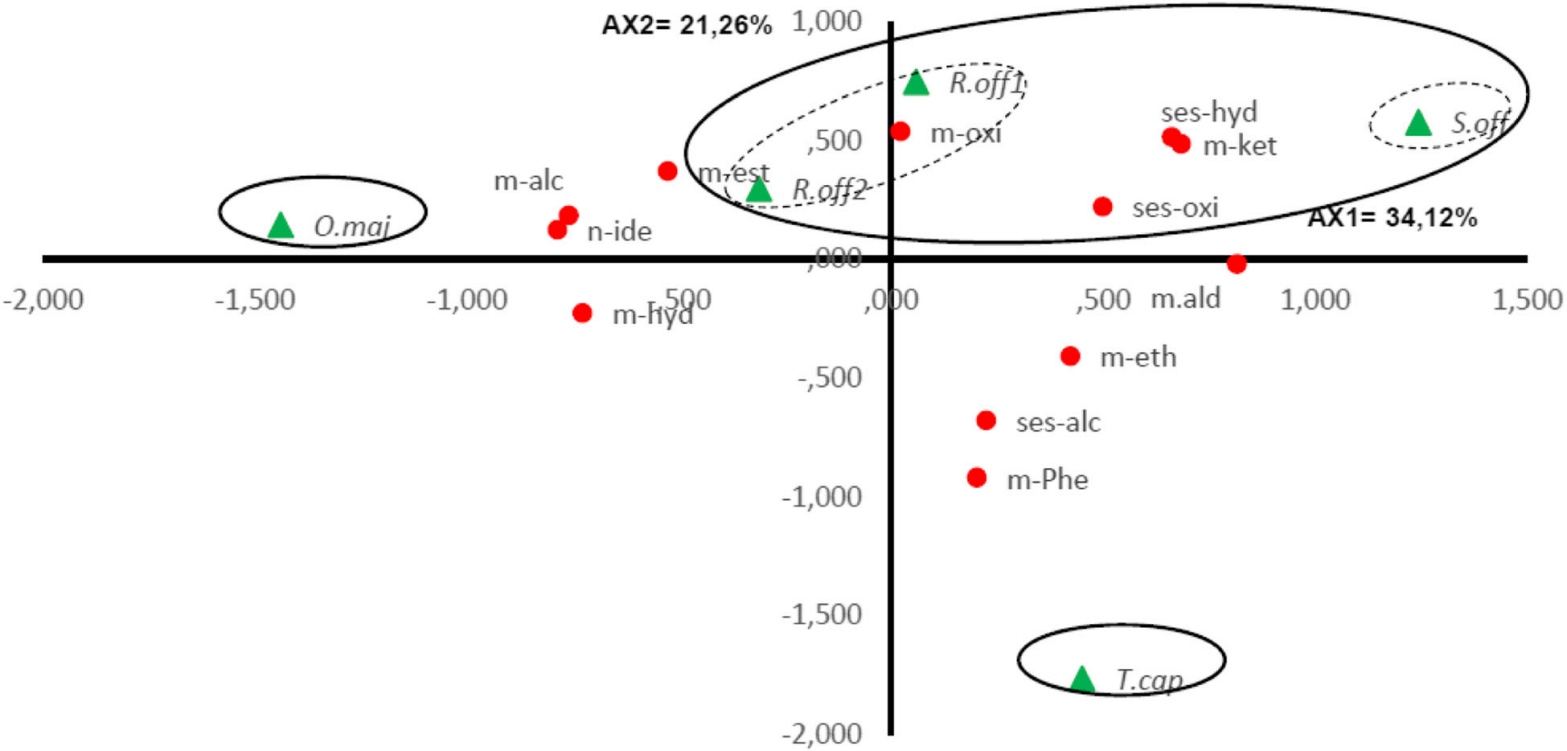
PCA of chemical classes for *S. officinalis, T. capitatus, R. officinalis*_*1*_, *R. officinalis2* and *O. majorana* essential oil. m-hyd, Monoterpene hydrocarbons; m-oxi, Monoterpene oxides; m-alc, Monoterpene alcohols; m-ket, Monoterpene Ketones; m-ald, Monoterpene aldehydes; m-est, Monoterpene esters; m-eth, Monoterpene ethers; m-phe, Monoterpene phenols, ses-hyd, Sesquiterpene hydrocarbons; ses-alc, Sesquiterpene alcohols; ses-oxi, Sesquiterpene oxides;n-ide, Not identified. For the Lamiceae species () abbreviations see Table 2

**Fig. 3:**
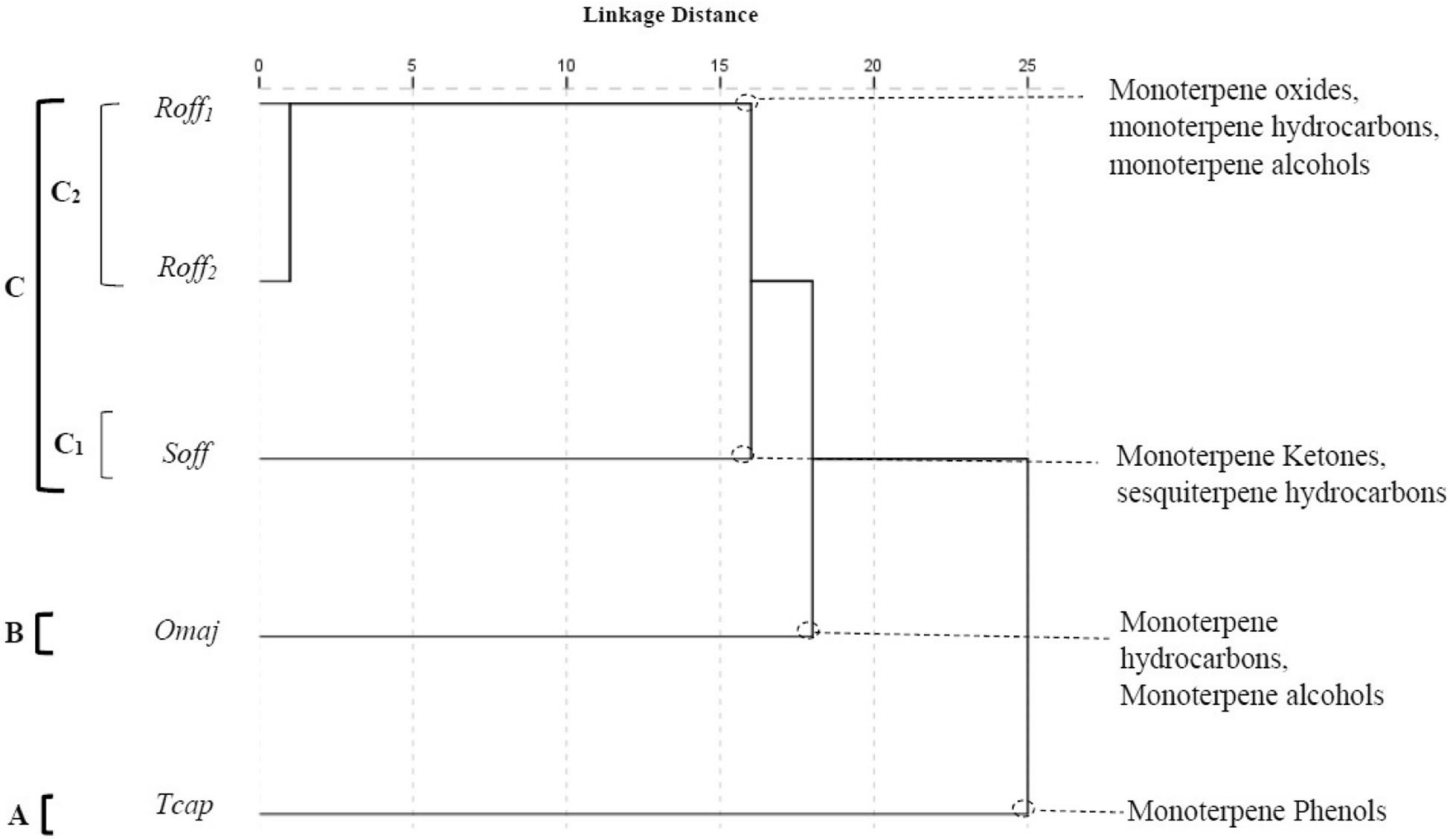
Dendrogram obtained by cluster analysis based on the Euclidian distances between groups of *S. officinalis, T. capitatus, R. officinalis*_*1*_, *R. officinalis2* and *O. majorana* Essential Oils. Classes that characterize the major subgroups are indicated. For the Lamiceae species () abbreviations see Table 2

The contents in different chemical classes for the five EOs was significantly different between the species (*p* < 0.05). The PCA horizontal axis explained 34.12% of the total variance and the vertical 21.26% (Fig. 2). The HCA (Fig. 3) based on the Euclidean distances between groups indicated the presence of three groups (*A*, *B* and *C*) identified by their contents in different chemical classes with a dissimilarity > 15. Group *C* was divided in two *Subgroups* (*C*_*1*_ and *C*_*2*_) with a dissimilarity > 5. The two species *T. capitatus* and *O. majorana* stand out in both HCA and PCA analyses, forming separate groups, group *A* and *B*, respectively. *Group A* formed by *T. capitatus* which EO was distinguished by the highest content in monoterpene phenols (56.6 ± 2.8%) and the smallest contents in monoterpene alcohols (4.6 ± 1.0%), monoterpene oxides (2.9 ± 4.6%) and monoterpene ketones (1.7 ± 2.8%). *Group B* was represented by *O. majorana* with an EO characterized by the highest contents in monoterpene hydrocarbons (40.9 ± 5.2%) and monoterpene alcohols (39.6 ± 3.1%) and the lowest content in sesquiterpene hydrocarbons (2.5 ± 0.6%). *S. officinalis* forming the *Subgroup C*_*1*_ was specified by the highest content in monoterpene ketones; however, it shared with the species of *Subgroup C*_*2*_ a relatively high content in sesquiterpene hydrocarbons (10.0 ± 3.3%). *R. officinalis*_1_ and *R. officinalis*_2_ constituting the *Subgroup C*_*2*_ were highlighted by the highest mean percentages of monoterpene oxides (47.2 ± 0.5 and 37.6 ± 0.5%) respectively and their poverty in the monoterpenic phenols and the sesquiterpenic alcohols. We noted also that *R. officinalis*_2_ and *O. majorana* EOs shared together the highest amount of monoterpene hydrocarbons.

### Major components

**Fig. 4:**
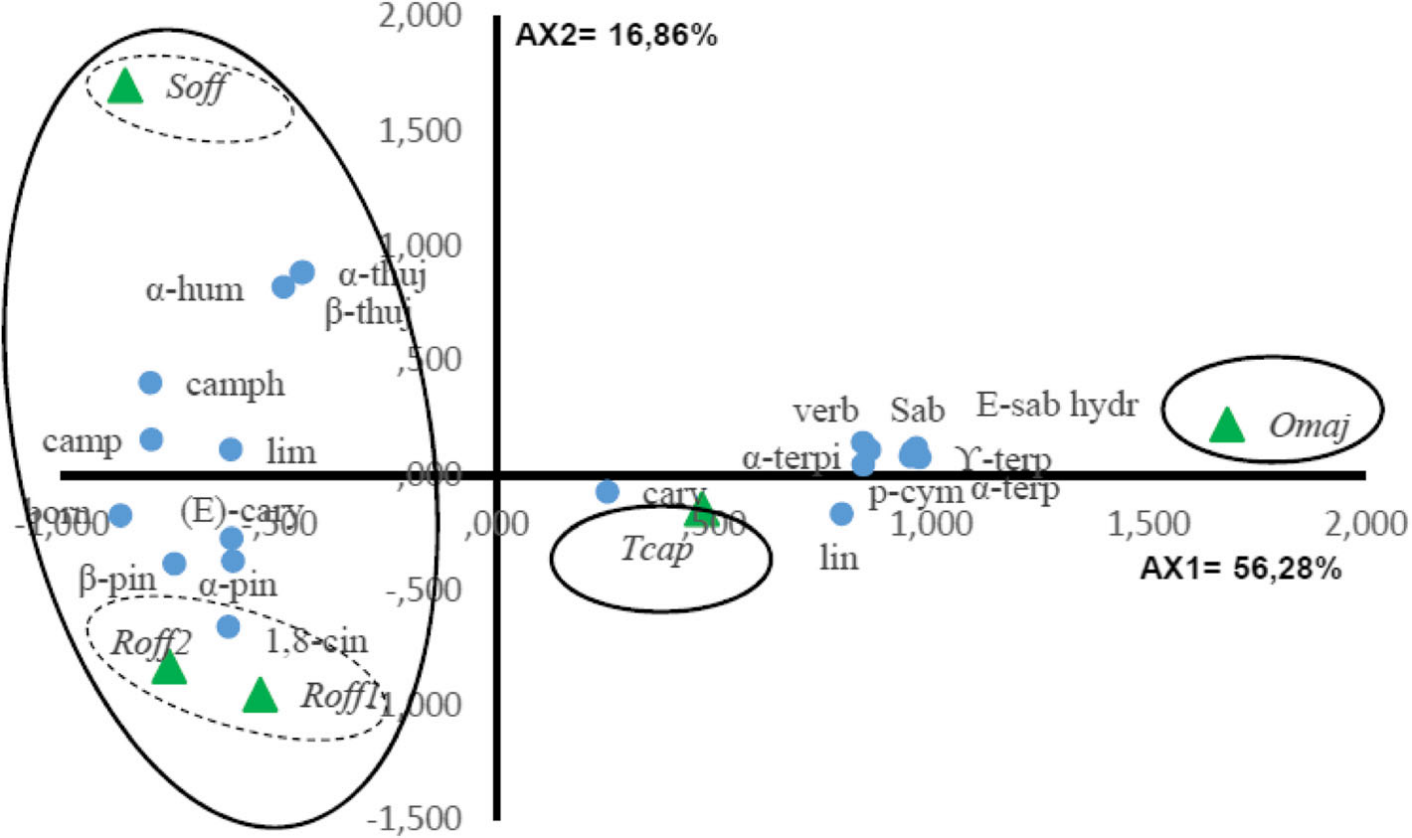
PCA of the 20 major components of *S. officinalis, T. capitatus, R. officinalis*_*1*_, *R. officinalis*_*2*_ and *O. majorana* essential oils. For the Lamiceae species () and components () abbreviations see Table 2

**Fig. 5:**
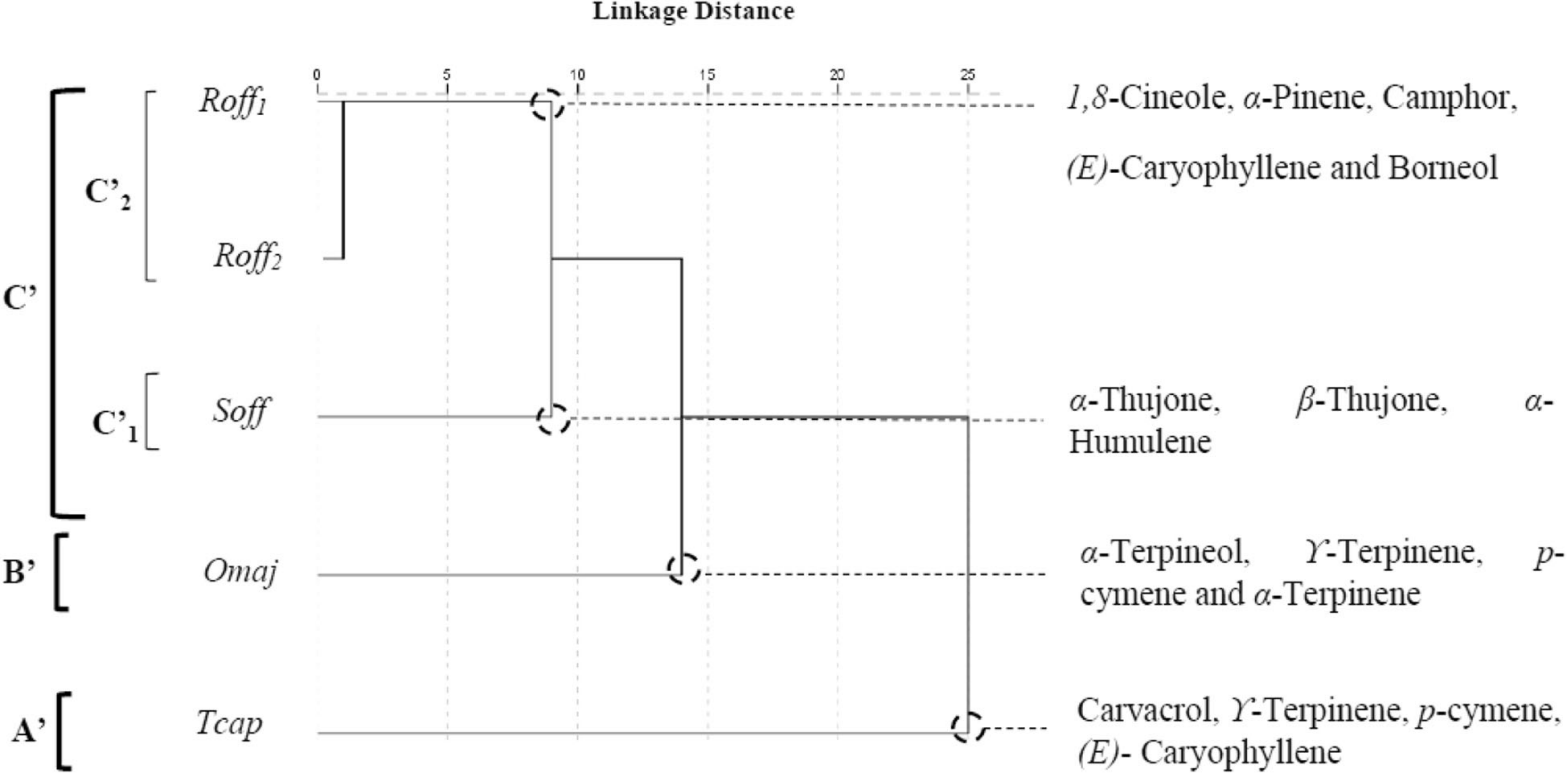
Dendrogram obtained by cluster analysis based on the Euclidean distances between groups of *S. officinalis, T. capitatus, R. officinalis1, R. officinalis2* and *O. majorana* essential oils. Components characterizing the major subgroups considered as chemotypes are indicated. For the Lamiceae species () and components () abbreviations see Table 2

Twenty components of the essential oils with a minimum content of 3.0% in at least one species were selected for the PCA and the HCA to evaluate whether they may be useful in reflecting the chemotaxonomic relationships in the four *Lamiaceae* species (Table 2). The contents of the selected oil components were significantly different between species (*p* < 0.05). The PCA horizontal axis explained 56.28% of the total variance while the vertical axis a further 16.86% (Fig. 4). The HCA based on the Euclidean distance between groups indicated three species groups (*A’, B′* and *C′*) identified by their essential oil chemotypes with a dissimilarity > 10 (Fig. 5) which was mainly due to the variation along the major axis in PCA analysis. With a dissimilarity > 5, group C′ was divided in two subgroups (C’1 and C’2) in both HCA and PCA analysis, which was affected essentially by the variation along the vertical axis.

**Table 2:** Content of S. officinalis, T. capitatus, R. officinalis_1_, R. officinalis2 and O. majorana Essential Oils in the 20 Compounds Selected for the Principal Component and the Hierarchical Cluster Analyses

Compound	Abreviation	content [%]				
		*S. officinalis*	*T. capitatus*	*R. officinalis*_*1*_	*R. officinalis*_*2*_	*O. majorana*
*α*-Pinene	*α*-pin	6.2 ± 3.0	0.8 ± 0.0	7.0 ± 3.9	19.4 ± 5.4	1.1 ± 0.3
Camphene	Camp	6.2 ± 1.4	0.2 ± 0.0	2.7 ± 1.4	6.5 ± 1.6	0.2 ± 0.0
Sabinene	Sab	0.1 ± 0.0	-^a)^	0.1 ± 0.0	Tr^b)^	2.2 ± 0.5
*β*-Pinene	*β*-pin	2.6 ± 0.5	0.5 ± 0.1	4.6 ± 1.4	3.6 ± 0.6	0.6 ± 0.1
*α*-Terpinene	α-terp	0.1 ± 0.2	2.2 ± 0.5	0.1 ± 0.1	0.4 ± 0.2	7.7 ± 1.3
*p*-Cymene	*p*-cym	2.1 ± 0.2	7.4 ± 0.8	1.9 ± 0.1	0.9 ± 1.0	9.7 ± 0.8
Limonene	Lim	1.6 ± 0.4	0.5 ± 0.4	–	2.5 ± 0.3	–
*1,8*-Cineole	*1,8*-cin	10.3 ± 1.4	2.9 ± 4.6	47.2 ± 0.5	37.6 ± 0.5	4.0 ± 0.5
*ϒ*-Terpinene	*ϒ*-terp	0.6 ± 0.0	10.8 ± 3.2	0.3 ± 0.0	0.5 ± 0.1	16.3 ± 1.5
*(E)*-Sabinene hydrate	E-sab hydr	–	1.1 ± 1.0	–	–	3.7 ± 0.2
Linalool	Lin	–	0.5 ± 0.9	1.0 ± 0.3	0.5 ± 0.1	2.8 ± 0.5
*α*-Thujone	*α*-thuj	22.8 ± 1.5	–	Tr	0.1 ± 0.0	–
*β*-Thujone	*β*-thuj	6.8 ± 0.5	–	0.1 ± 0.0	Tr	–
Camphor	Camph	19.3 ± 2.1	1.6 ± 2.7	13.3 ± 2.9	7.1 ± 1.3	Tr
Borneol	Born	4.0 ± 0.4	1.0 ± 0.8	5.4 ± 1.5	4.4 ± 1.2	–
*α*-Terpineol	*α*-terpi	0.1 ± 0.0	0.2 ± 0.4	2.6 ± 0.7	1.6 ± 0.6	27.4 ± 2.8
Verbenone	Verb	–	–	Tr	Tr	3.7 ± 0.5
Carvacrol	Carv	Tr	56.1 ± 3.0	–	–	0.6 ± 0.4
*(E)*-Caryophyllene	*(E)*-cary	3.9 ± 1.3	4.2 ± 0.3	5.2 ± 0.9	5.0 ± 3.1	1.7 ± 0.5
*α*-Humulene	*α*-hum	4.8 ± 2.0	0.2 ± 0.0	0.3 ± 0.4	0.7 ± 0.4	0.1 ± 0.0

^a^-: not detected. ^b^*Tr* Trace (< 0.1%)

The *T. capitatus* EO of Group A’ stand out forming separate group in both PCA and HCA analysis and was characterized by the highest content of carvacrol (56.1 ± 3.0%) and a relatively high contents of *p*-cymene (7.4 ± 0.8%) and *ϒ*-terpinene (10.8 ± 3.2%). However, it was very poor in *1,8*-cineole (2.9 ± 4.6%), *α*-pinene (0.8 ± 0.0%) and camphene (0.2 ± 0.0%). Group B’ correlated positively with axis1 and was represented by *O. majorana*, which EO was characterized by the highest contents of *α*-terpineol (27.4 ± 2.8%), *ϒ*-terpinene (16.3 ± 1.5%), *p*-cymene (9.7 ± 0.8%), *α*-terpinene (7.7 ± 1.3%), and by the lowest content of the compounds which were negatively correlated with axis 1, such as *α*- humulene and camphor (tr) .

*Subgroup C’*_*1*_ was represented by *S. officinalis*, which EO was characterized by the highest contents of *α*-thujone (22.8 ± 1.5%), *β*-thujone (6.8 ± 0.5%) and *α*-humulene (4.8 ± 2.0%).

*R. officinalis*_1_ and *R. officinalis*_2_ constituted the *subgroup C’*_*2*_. Their EOs contained the highest mean percentage in *1,8*-cineole (47.2 ± 0.5 and 37.6 ± 0.5%, resp.) compared to the moderate level (10.3 ± 1.4%) of this component in *S. officinalis*. They are characterized by a lower content of camphor (13.3 ± 2.9 and 7.1 ± 1.3%) in comparison with *S. officinalis* camphor content (19.3 ± 2.1%). The Group C′ EOs shared together the presence of the highest mean percentages of *(E)*-caryophyllene (5.2 ± 0.9, 5.0 ± 3.1 and 3.9 ± 1.3%) and *β*-pinene (4.6 ± 1.4, 3.6 ± 0.6 and 2.6 ± 0.5%) for *R. officinalis*_1_, *R. officinalis*_2_ and *S. officinalis* EOs respectively. The separation of the two *Rosmarinus* oil samples was essentially due to the higher content of α-pinene in *R. officinalis*_2_ EO (19.4 ± 5.4%) versus (7.0 ± 3.9%) for that of *R. officinalis*_1_.

### Antibacterial activity

The essential oils were tested for their supposable antibacterial activity against five model bacteria. Antibacterial effects are reported as inhibition zones, and “in vitro activity” as MIC and MBC. The results obtained using the disc diffusion method recorded in MH agar were summarized in Table 3. According to the value of the inhibition zone diameter (IZD) expressed in mm, results were appreciated as follows: not sensitive (−) for diameter equal to 8 mm or below; sensitive (+) for diameter between 8 and 14 mm; very sensitive (++) for diameter 14 and 20 mm and extremely sensitive (+++) for diameter equal or larger than 20 mm [34, 35].

**Table 3:** Antibacterial activity (Diameter of the inhibition zone) of the 5 Tunisian Lamaiaceae plants essential oils and antibiotic *)

Essential oils	Microorganisms				
	*E. coli* *Ecol*	*S. enterica* *Sent*	*P. aeruginosa* *Paer*	*B. subtilis* *Bsub*	*S. aureus* *Saur*
*S. officinalis*	11.3^a^ ± 1.2	7.7^a^ ± 1.2	7.0^a^ ± 1.0	17.3^b^ ± 0.6	8.3^a^ ± 0.6
*T. capitatus*	20.3^d^ ± 0.6	20.0^d^ ± 1.0	20.3^d^ ± 0.6	30.0^e^ ± 1.0	15.0^c^ ± 1.0
*R. officinalis*_*1*_	11.0^a^ ± 1.0	8.7^a^ ± 0.6	8.3^b^ ± 0.8	19.3^c^ ± 1.2	10.0^ab^ ± 1.7
*R. officinalis*_*2*_	15.3^b^ ± 0.6	7.7^a^ ± 1.2	6.0^a^ ± 0.0	25.3^d^ ± 0.6	10.3^ab^ ± 0.6
*O. majorana*	14.0^b^ ± 1.0	14.0^b^ ± 1.0	6.3^a^ ± 0.6	6.0^a^ ± 0.0	12.0^b^ ± 1.0
*Gentamicine®*	17.3^c^ ± 0.6	17.3^c^ ± 0.6	16.0^c^ ± 1.0	24.3^d^ ± 1.2	21.7^d^ ± 1.5

*Values are means (mm. ± SD) of the triplicate determination

*Values with different letters differ significantly by SNK test (p < 0.05)

To determine the antibacterial power of the studied EOs, the MBC/MIC ratio was calculated and interpreted based on the classification of Schwarz et al. (2010). The EO is considered bactericidal when this ratio is lower or equal to 4 whereas it’s bacteriostatic when it’s greater than 4 [36].

To evaluate the correlations between the antibacterial activities and the essential oils, all the mean values of the inhibition zone diameters were subjected to PCA and HCA analysis.

**Fig. 6:**
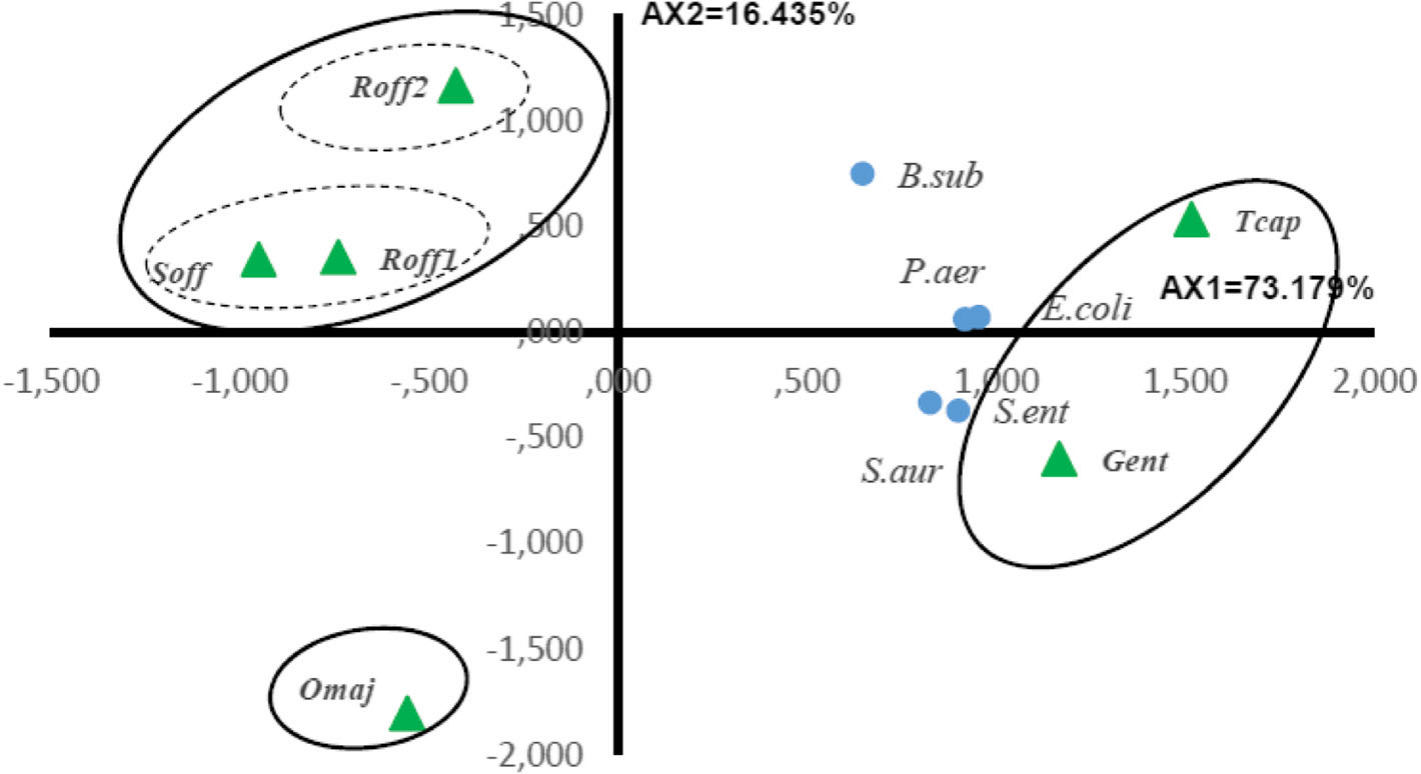
PCA of the antibacterial activity of *S. officinalis, T. capitatus, R. officinalis*_*1*_, *R. officinalis*_*2*_ and *O. majorana* essential oils against five bacterial strains. For the Lamiceae species () abbreviations see Table 2. For the bacterial strains () abbreviations see Table 3

**Fig. 7:**
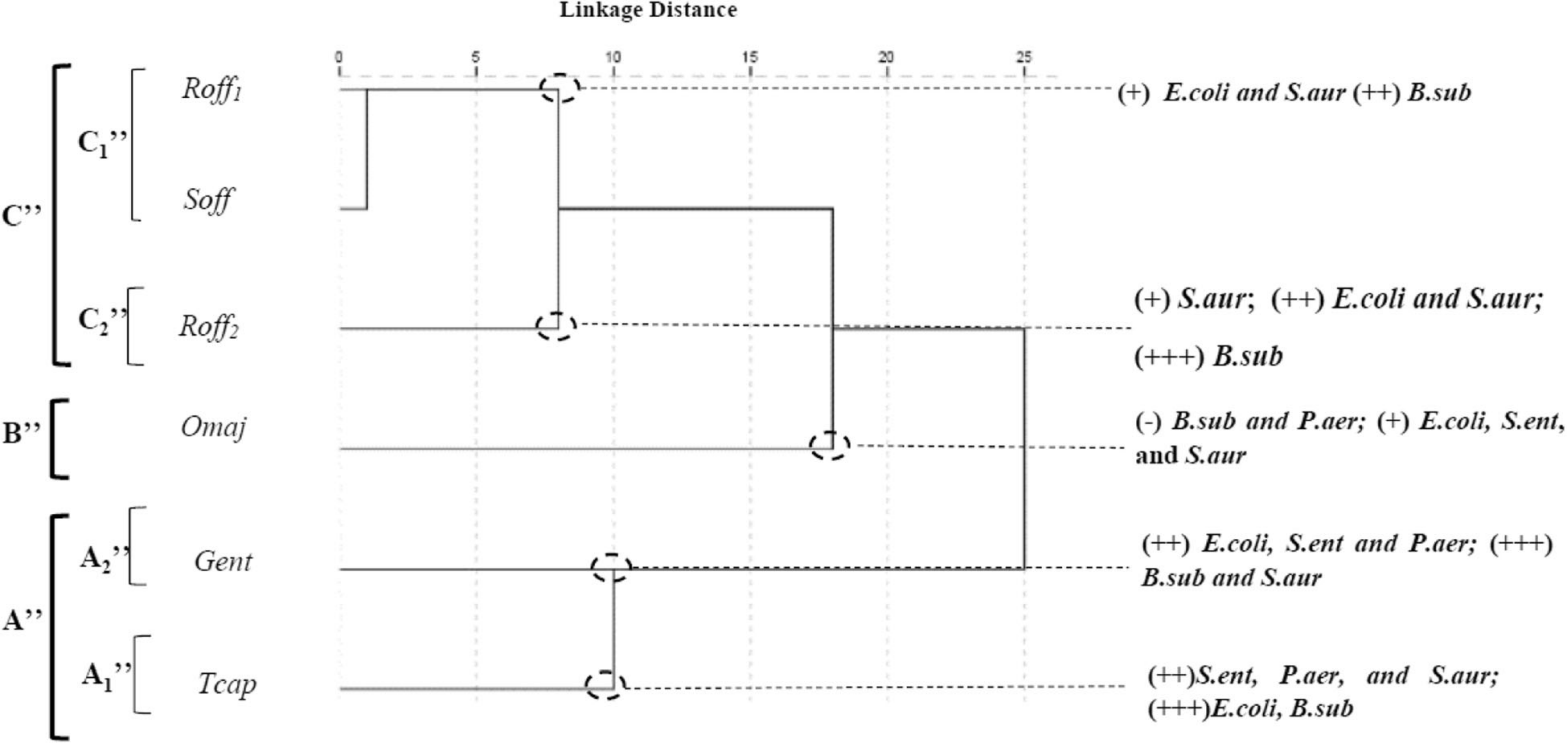
Dendrogram obtained by hierarchical cluster analysis based on the Euclidean distance between groups of the antibacterial activities of *S. officinalis, T. capitatus, R. officinalis*_*1*_, *R. officinalis*_*2*_ and *O. majorana* essential oils. For the Lamiceae species () abbreviations see Table 2. For the bacterial strains () abbreviations see Table 3

The PCA horizontal axis explained 73.18% of the total variance while the vertical axis a further 16.43% (Fig. 6). The HCA based on the Euclidean distances between groups indicated 3 groups (A″, B″ and C″) of species identified by their antibacterial inhibition growth with a dissimilarity ⩾10. The first principal axis separated the groups A″ and C″, whereas the second principal axis allowed the separation of the group B″ from the groups C″ in the ACP (Fig. 6) and the HCA (Fig. 7).

Group A″ was constituted by the standard antibiotic Gentamicin® and *T. capitatus*, which were characterized by being very active or extremely active against all the tested strains (15.0 ± 1.0 < IZD < 30.0 ± 1.0 mm). With a dissimilarity > 5, group A″ was divided in two subgroups (A″_1_ and A″_2_). *B. subtilis* was extremely sensitive to both *T. capitatus* and Gentamicine® forming respectively *Subgroup A″*_*1*_ and *Subgroup A″*_*2*_. *T. capitatus* was characterized by being extremely active against *E. coli* (20.3 ± 0.6 mm), *S. enterica (20.0 ± 1.0 mm)* and *P. aeruginosa* (20.3 ± 0.6 mm) and very active against *S. aureus* (15.0 ± 1.0 mm) while Gentamicine® was extremely active against this last strain and very active against the three first ones.

Group B″ was constituted by *O. majorana*, it stands out forming a separate group in HCA and PCA and correlated negatively with the two principle axis, it was characterized by being inactive against *B. subtilis* (6.0 ± 0.0 mm) and moderately active against *E. coli*, *S. enterica* and *S. aureus* (14.0 ± 1.0, 14.0 ± 1.0, 12.0 ± 10 mm, respectively).

Group C″ was divided in two *Subgroups C″*_*1*_ represented by *R. officinalis*_1_ and *S. officinalis*, and *Subgroup C″*_*2*_ represented by *R. officinalis*_2_. Group C″ species were characterized by being inactive or moderately active against *S. enterica* (7.7 ± 1.2, 8.7 ± 0.6, 7.7 ± 1.2 mm) and *P. aeruginosa* (7.0 ± 1.0, 8.3 ± 0.8, 6.0 ± 0.0 mm), but active against *S. aureus* (8.3 ± 0.6, 10.0 ± 1.7, 10.3 ± 0.6 mm). Subgroup *C″*_*2*_ represented by *R. officinalis*_2_ oil was characterized by being extremely active against *B. subtilis* (25.3 ± 0.6 mm) and very active against *E. coli* (15.3 ± 0.6 mm) in comparison with Subgroup C″_1_ species (17.3 ± 0.6–19.3 ± 1.2 mm; 11.0 ± 1.0–11.3 ± 1.2 mm; resp.).

Besides,

Table 4 summarizes the MIC and MBC values of the tested Lamiaceae EOs obtained by the dilution method in MH broth. *T. capitatus* showed the most important bactericidal effect against all the strains (MIC range: 0.73–2.94 mg mL^− 1^; MBC range: 0.73–2.94 mg mL^− 1^) while *R. officinalis*_*2*_ exhibited the weaker antibacterial activity against the tested strains (MIC range: 45.50–91.00 mg mL^− 1^; MBC range: 91.00–182.00 mg mL^− 1^).

**Table 4:** Minimal inhibition concentration (MIC), minimal bactericidal concentration (MBC) and ration MBC/MIC of the aerial parts essential oils extracted from 05 Tunisian Lamiaceae plants against 5 bacterial strains

Essential oils	Tested microorganisms				
	*E. coli*^a)^	*S. ent*	*P. aer*	*B. sub*	*S. aur*
*S. officinalis*					
MIC	22.78^b)^	22.78	22.78	22.78	22.78
MBC	45.55	22.78	22.78	22.78	22.78
MBC/MIC	**2.00**	**1.00**	**1.00**	**1.00**	**1.00**
*T. capitatus*					
MIC	0.73	0.73	1.47	2.94	0.73
MBC	0.73	0.73	2.94	2.94	0.73
MBC/MIC	**1.00**	**1.00**	**2.00**	**1.00**	**1.00**
*R. officinalis*_*1*_					
MIC	11.38	22.75	22.75	22.75	22.75
MBC	22.75	22.75	45.50	22.75	45.50
MBC/MIC	**2.00**	**1.00**	**2.00**	**1.00**	**2.00**
*R. officinalis*_*2*_					
MIC	45.50	45.50	91.00	91.00	45.50
MBC	182.00	91.00	182.00	91.00	91.00
MBC/MIC	**4.00**	**2.00**	**2.00**	**1.00**	**2.00**
*O. majorana*					
MIC	45.00	22.50	45.00	22.50	22.50
MBC	45.00	22.50	45.00	22.50	22.50
MBC/MIC	**1.00**	**1.00**	**1.00**	**1.00**	**1.00**

^a^*E. coli = Escherichia coli CIP 53.126; S. ent = Salmonella enterica CIP80.39;*

*P. aer = Pseudomonas aeruginosa CIP 82118; B. sub = Bacillus subtilis CIP 52.62; S. aur = Staphylococcus aureus CIP 4.83.*

^b^Values are expressed as mg/mL

## Discussion

### Chemical composition

Tunisian *S. officinalis* EO have nearly similar composition to that obtained by Gomes et al. (2001) and Bouaziz et al. (2009) [37, 38] and disagree with Hayouni et al. (2008) who reported that *1,8*-cineole (33.27%) was the major compound followed by *β*-thujone (18.40%), *α*-thujone (13.45%), borneol (7.37%) and camphor (3.31%) [13].

*T. capitatus* investigated EO was characterized by a higher amount in monoterpene hydrocarbons than that noted by Russo et al. (2013) [39] and Jemaa et al. (2018) [40] (9.56–13.15%), and (15.10%) respectively. Their EOs were richer in carvacrol (76.79 and 76.1%; resp.) but poor in *ϒ*-terpinene (3.71 and 6.7%; resp.) in comparison with our finding. El Jalel et al. (2018) found that the main constituents of *T. capitatus* aerial part EO from Libya were carvacrol (58.56%), which was comparable to our finding, while *β*-caryophyllene (7.41%), ledene (6.57%), caryophyllene oxide (6.26%) and *α*-humulene (5.20%) contents were higher than that obtained in our results [41]. We noticed the presence of a relatively high content of *1,8*-cineole (2.9%) in comparison with the cited studies.

*O. majorana* EO content in monoterpene hydrocarbons (40.9%) and oxygenated monoterpenes (51.5%) was comparable to the results of Hajlaoui et al. (2016) [22] while El Akhal et al. (2014) showed a higher content in monoterpene hydrocarbons (51.7%) and a lower content in oxygenated monoterpene (44.38%) [42]. Moreover, Hajlaoui et al. (2016) and Mossa et al. (2011) characterized different major compound such as terpinene-*4*-ol (23.2, 29.97%; resp.) [21] [22]. However, *ϒ*-terpinene was the second most important component with relatively comparable amounts (15.4, 18.57 and 10.5%).

Results obtained for *R. officinalis* EOs were in accordance with previous studies reporting that *1,8*-cineole (37.6 and 47.2%), camphor (7.1 and 13.3%) and *α*-pinene (7.0 and 19.4%) were the major components of *R. officinalis* EO [43, 44]while Verbenone (4.4–24.9%) was found to be the major component of *R. officinalis* EO from Corsica and Sardinia [45].

### Antibacterial activity

The essential oils antibacterial activity showed considerable variation among the different Lamiaceae species and bacterial strains. *T. capitatus* EO antibacterial activity was even higher than that of the standard antibiotic Gentamicine®. The IZD of *T. capitatus* ranged from (15.0 ± 1.0 to 30.0 ± 1.0 mm) and MIC ranged from (0.73 to 2.94 mg/mL). In fact, *T. capitatus* EO was characterized by the most important mean percentage of the monoterpenoid phenol carvacrol (56.1 ± 3.0%), and important contents of hydrocarbon monoterpenes *p*-cymene (7.4 ± 0.8%) and *ϒ*-terpinene (10.8 ± 3.2%). Previous study proved that the thyme essential oil activity was mainly due to the presence of phenolic compounds and monoterpene hydrocarbons [46]. It was reported that an antimicrobial action of phenolic compounds was related to the inactivation of certain cellular enzymes. It depends on membrane permeability changes and consequently on the rate of penetration into the cell [39]. Increased membrane permeability is a major factor in the mechanism of antimicrobial action of phenolic compounds, which may disrupt membranes and cause a loss of cellular integrity and eventual cell death.

Activity amongst the phenols and alcohols is at least partly due to the hydroxyl group, which has intrinsic antimicrobial activity and contributes to the relatively greater solubility of these components in biological membranes. Furthermore, the ability of components to release or accept protons has been postulated as an important factor in antimicrobial activity [5, 47, 48].

Another study attributed the activity of carvacrol to its hydroxyl group. In fact, they confirmed the importance of this group in term of activity in the phenolic structure when they compared carvacrol to its methyl ether [49] it was demonstrated in the same study that *p*-Cymene, the third major element according to percentage, does not show antibacterial efficacy when used alone. Moreover, other studies [50, 51] have shown that EOs exhibit stronger antimicrobial activity than that of their major constituents or their mixtures which suggests synergistic effects of the minor components, but also the importance of all components in relation to the biological activity of EOs.

The activity was decreased with *O. majorana* oil with MIC values ranging from 22.5 to 45.0 mg/mL. This EO contained a very lower content of carvacrol and higher mean percentage of p-cymene and *ϒ*-terpinene. *O. majorana* oil was also characterized by the highest amount of monoterpene hydrocarbons, and alcohol monoterpene α-terpineol. The combination of these results permitted as to deduce that the main antibacterial activity against the studied strains might be attributed to the richness of the essential oils in the phenol carvacrol.

It is interesting to note the susceptibility to the *T. capitatus* EO of *P. aeruginosa*, which is known to be a very resistant bacterium even to synthetic drugs, with MICs ranging from 0.73 to 2.94 mg/mL. This result confirm those found with EL Jalel et al. (2018) [41].

*S. officinalis*, *R. officinalis*_1_ and *R. officinalis*_2_ were placed in the same group in both antibacterial ACP and HCA analyses, but within chemical ACP and HCA analysis, they were classified in two different subgroups. *S. officinalis* was characterized by the most important amount in monoterpene ketones (48,9 ± 4.1%) while *R. officinalis* were characterized by the highest amounts of monoterpenes oxides (47.2 ± 0.5 and 37.6 ± 0.5%).

These results suggested that the major components from these two chemical classes could be responsible for producing a similar inhibition. Our results were confirmed by Hammer et al. who demonstrated that camphor and *1,8*-cineole were the principle components responsible for the antibacterial activity against *B. subtilis*, *E. coli* and *S. aureus* [5]. In the same way, Longaray Delamare et al. (2007) attributed the antimicrobial activities of *S. officinalis* against *E. coli*, *P. aeruginosa*, *B. subtilis* and *S. aureus* to the presence of high concentrations of thujone, *1,8*-cineole and camphor [17].

The *R. officinalis* EOs was noted to be active against all the bacterial strains but in different degrees. In fact, *R. officinalis*_*1*_ was more potent with MICs values ranging from 11.38 mg/mL to 22.75 mg/mL when *R. officinalis*_*2*_ MICs ranged from 45.00 mg/mL to 91.00 mg/mL. These results could be explained by the difference in the mean percentages of some components in these EOs. Actually, *R. officinalis*_*1*_ EO was richer in oxygenated monoterpene active compounds such as *1,8*- cineole (47.2 ± 0.5% Vs. 37.6 ± 0.5%) and camphor (13.3 ± 2.9% Vs. 7.1 ± 1.3%). However, *R. officinalis*_*2*_, was characterized by higher amounts of hydrogenated monoterpene compounds, like *α*- pinene (19.4 ± 5.4% Vs. 7.0 ± 5.4%) and camphene (6.5 ± 1.6% Vs. 2.7 ± 1.4%). These finding confirm the results of Griffin et al. (1999) and Van de Vel et al. (2017). These authors suggested that the water solubility and the hydrogen bonding capacity are the main factors influencing MIC values of terpenoids against *P. aeruginosa*, *S. aureus*, and *E. coli* [52, 53], since *1,8*- cineole and camphor are distinguished by a higher water solubility and hydrogen bonding capacity than *α*- pinene and camphene. Thereby, we can confirm that the qualitative and quantitative differences between *R. officinalis* EOs chemical composition could affect their antibacterial activity [54].

The MBC/MIC ratio ranged from 1 to 4 for all the EOs, indicating a bactericidal activity for these lamiaceae EOs against the tested bacterial strains. Other Moroccan studies showed that *R. officinalis* had a bacteriostatic effect on *S. aureus* [44], whereas *O. majorana* had a bactericidal effect on *E. coli* and was bacteriostatic on *S. aureus* and *B. subtilis* [55]. The variation in the EOs antibacterial activity could be attributed to the variation of the chemical composition, which is depending of some factors including species, the plant origin, climatic conditions, distillation conditions, adaptive metabolism of plant [56, 57] as well as to the bacterial strains susceptibility [58].

## Conclusion

The PCA and the HCA of the chemical composition of the studied Lamiaceae EOs separated them in four groups and sub-groups with different chemotypes. *T. capitatus* was the richest species in carvacrol, *S. officinalis* was the richest species in α-thujone, and *R. officinalis*_1_ was the richest species in 1,8-cineole. The EOs antibacterial activity varied significantly within species and within strains. In general, EOs have shown promising antibacterial activities we noted a pronounced activity of *T. capitatus* against *P. aeruginosa*. These results may represent useful and valuable leads for the development of new disinfectant products.

## Data Availability

Data and materials are available from authors on reasonable request.

## Abbreviations

*R. officinalis*: *Rosmarinus officinalis*
*T. capitatus*: *Thymus capitatus*
*O. majorana*: *Origanum majorana*
*S. officinalis*: *Salvia officinalis*
GC: Gaz chromatography
GC/MS: Gaz chromatography coupled to the mass spectroscopy
HP: Hewlett-Packard
FID: Flame ionization detector
MH: Mueller–Hinton
MIC: Minimal inhibition concentration
MBC: Minimum bactericidal concentration
IZD: Inhibition zone diameter
DMSO: Dimethyl sulfoxide
PCA: Principal components analysis
HCA: Hierarchical clusters analysis
RI: Retention index
Gram(−): Gram negative
Gram(+): Gram positive
